# Characterization of PetM cytochrome *b6f* subunit 7 domain-containing protein in tomato

**DOI:** 10.1093/hr/uhad224

**Published:** 2023-11-08

**Authors:** Mustafa Bulut, Adriano Nunes-Nesi, Alisdair R Fernie, Saleh Alseekh

**Affiliations:** Root Biology and Symbiosis, Max-Planck-Institute of Molecular Plant Physiology, Am Mühlenberg 1, 14476 Potsdam, Germany; Departamento de Biologia Vegetal, Universidade Federal de Viçosa, Viçosa 36570-900 MG, Brazil; Root Biology and Symbiosis, Max-Planck-Institute of Molecular Plant Physiology, Am Mühlenberg 1, 14476 Potsdam, Germany; Plant Metabolomics, The Center for Plant Systems Biology and Biotechnology, 4000 Plovdiv, Bulgaria; Root Biology and Symbiosis, Max-Planck-Institute of Molecular Plant Physiology, Am Mühlenberg 1, 14476 Potsdam, Germany; Plant Metabolomics, The Center for Plant Systems Biology and Biotechnology, 4000 Plovdiv, Bulgaria

## Abstract

In recent years, multiple advances have been made in understanding the photosynthetic machinery in model organisms. Knowledge transfer to horticultural important fruit crops is challenging and time-consuming due to restrictions in gene editing tools and prolonged life cycles. Here, we characterize a gene encoding a PetM domain-containing protein in tomato. The CRISPR/Cas9 knockout lines of the PetM showed impairment in the chloroplastic electron transport rate (ETR), reduced CO_**2**_ assimilation, and reduction of carotenoids and chlorophylls (Chl) under several light conditions. Further, growth-condition-dependent elevation or repression of Chl *a/b* ratios and de-epoxidation states were identified, underlining possible impairment compensation mechanisms. However, under low light and glasshouse conditions, there were basal levels in CO_**2**_ assimilation and ETR, indicating a potential role of the PetM domain in stabilizing the cytochrome *b6f* complex (C*b6f*) under higher light irradiance and increasing its quantum efficiency. This suggests a potential evolutionary role in which this domain might stabilize the site of the C*b6f* regulating ratios of cyclic and linear electron transport and its potential importance during the conquest of terrestrial ecosystems during which plants were exposed to higher irradiance. Finally, the results are discussed with regard to metabolism and their implication to photosynthesis from an agronomic perspective.

## Introduction

Photosynthesis in terrestrial plants, microalgae, and cyanobacteria involves two distinct photosystems, photosystem I (PSI) and photosystem II (PSII). Following the absorption and transfer of light energy by light-harvesting complexes to the photosystem reaction centers, a sequence of electron transport processes occurs to generate Adenosine triphosphate (ATP) and the reduced form of nicotinamide-adenine dinucleotide phosphate (NADPH) for carbon dioxide (CO_2_) assimilation. The process of photosynthetic electron transport comprises two main pathways, the linear electron transport (LET), which extends from water to NADPH production, and the cyclic electron transport (CET) around PSI (known as PSI-CET), involving the recycling of reduced ferredoxin (Fd) or NADPH to the intersystem chain [1, 2]. In green plants, LET characterized by three prominent membrane-bound complexes, namely PSII, C*b6f*, and PSI. Within this framework, small mobile electron carriers shuttle electrons between these three complexes [1–3]. Additionally, PSI-CET includes C*b6f*, PSI, the chloroplast NADPH dehydrogenase-like complex, and proton gradient regulation proteins, such as PGR5 or PGR5-like1 [4–6]. C*b6f* plays a pivotal role in connecting both LET and CET by acting as a plastoquinol–plastocyanin oxidoreductase, transferring electrons from plastoquinol to plastocyanin. This electron transfer event is accompanied by the movement of protons across the thylakoid membrane, creating a trans-thylakoid membrane proton gradient (ΔpH), which drives ATP synthesis [1, 7]. Notably, the C*b6f* complex, in conjunction with the Stt7 kinase, modulates the sizes of the light-harvesting antennae of PSI and PSII through state transitions mediated by reversible phosphorylation of light-harvesting complex II. These transitions are influenced by the redox state of the plastoquinone pool [7, 8]. Furthermore, the abundance of C*b6f* serves as a molecular target for dynamic long-term acclimation, enabling the regulation and optimization of photosynthesis in response to changing environmental conditions [7–9].

Whilst the content of C*b6f* highly depends on environmental conditions as well as developmental stage in higher plants and increases with actinic light intensities, the amplitude of the change is species-dependent. Enzymatic, the Rubisco content and Calvin–Benson–Bassham cycle activity are strongly co-regulated by C*b6f* [10]. Further, loss of C*b6f* foreshadows the degradation of PSII, PSI and chloroplastic ATP synthase in *Phaseolus vulgaris* [11, 12] and decreased assimilation capacity in aging leaves is tightly correlated to the ontogenic loss of C*b6f* [13, 14]. Structurally, the CB6F is comprised of eight subunits, but only the small PETM subunit and the Rieske FeS protein (PETC) are nuclear-encoded [15]. The four small subunits are of 3 to 4 kDa size and are bound to each monomer of the C*b6f* [16]. PetG and PetN are essential for C*b6f* assembly and stability as demonstrated [17], while the other two small subunits PetL and PetM are located peripheral of the C*b6f* [18]. Multiple discoveries were archived in recent years, by the characterization of genes involved in the assembly of photosynthetic complexes as well as photosynthetic regulation under controlled and fluctuating light conditions [19–22]. Among those, the PETM subunit was shown in recent studies to play an essential role in CB6F assembly and stability thereby ensuring electron transport between photosystems II and I [23]. The massive reduction of C*b6f* complex content in tobacco also resulted in a massive impairment of thylakoid membrane energization and thylakoid lumen acidification, and therefore of photoprotective non-photochemical quenching (NPQ). Impaired NPQ together with a massive over-reduction of the PSII acceptor side resulted in strong PSII photoinhibition, massive generation of reactive oxygen species (ROS) and photobleaching of leaves. In planta, the two studies assessing PETM function were performed in the model plants *Nicotiana tabacum* [23] and *Arabidopsis thaliana* [24]. However, the role of this protein has not been characterized in horticultural fruit crop species, such as *Solanum lycopersicum*. In particular, studies giving insights by integration of the alteration in their photosynthetic capacities, metabolism and yield characteristics under varying environments are rare.

**Figure 1:**
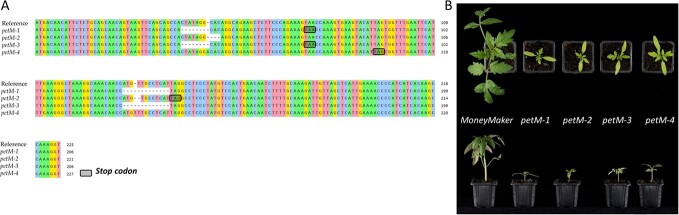
DNA sequence comparison of the CRISPR/Cas9 generated lines**. (A)** Morphological phenotypes of *petM* mutant lines and “MoneyMaker” during early cultivation at 150 μmol photons m^−2^ s^−1^. **(B)** CRISPR/Cas9 mediated PetM editing in each independent line compared to their reference sequence. The black boxes indicate early stop codons originating from Indels.

Interestingly, according to the tomato genome (*S. lycopersicum* NCBI annotation release 103) there are two alleles predicted, one on chromosome 1 and one on chromosome 10, to contain the PetM domain. These two alleles vary in their expression levels [25]. Here, we investigated the major homolog of the plastoquinol-plastocyanin oxidoreductase subunit 7 (*Solyc01g109040)* which uniquely shares the same orthogroup with in A. thaliana AT2G26500). The protein sequence contains a PetM domain of C*b6f* subunit 7, which orthologues in *A. thaliana* and *Nicotiana tabacum* were previously characterized as essential genes in photosynthesis [23, 24] as well as a glycoside hydrolase, family 7 domain at the N-terminal transit peptide site. It was shown in Arabidopsis that fusion of the 84 N-terminal amino acids leads to targeting to the chloroplast [24]. In this work, the CRISPR technique was used to generate gene-edited tomato lines. The results showed that unlike the Arabidopsis and Tobacco, the tomato CRISPR/Cas9 PetM knockout (KO) plants were able to recover from the photoinhibition. Further, metabolic changes were demonstrated especially in their lipidomic profiles. Overall, the tomato KO lines exhibited delayed growth throughout their development with later setting fruits. Given the relative lack of importance of this subunit in cyanobacteria *Synechocystis* PCC 6803 [26] and its peripheral binding position in the CB6F, where 9-cis-β-carotene and thylakoid soluble phosphoprotein (TSP9) are located [20], indicates the presence of a potential alternate route to retain photonic energy capture. Evolutionary, this might play a role in the adaptation to optimizing energy capture when plants were exposed to higher irradiance during the conquest of terrestrial ecosystems. Ultimately, the current study demonstrates photosynthetic and metabolic shifts upon changing growth conditions and developmental stages, and additional yield deficits of *petM* mutants.

**Figure 2:**
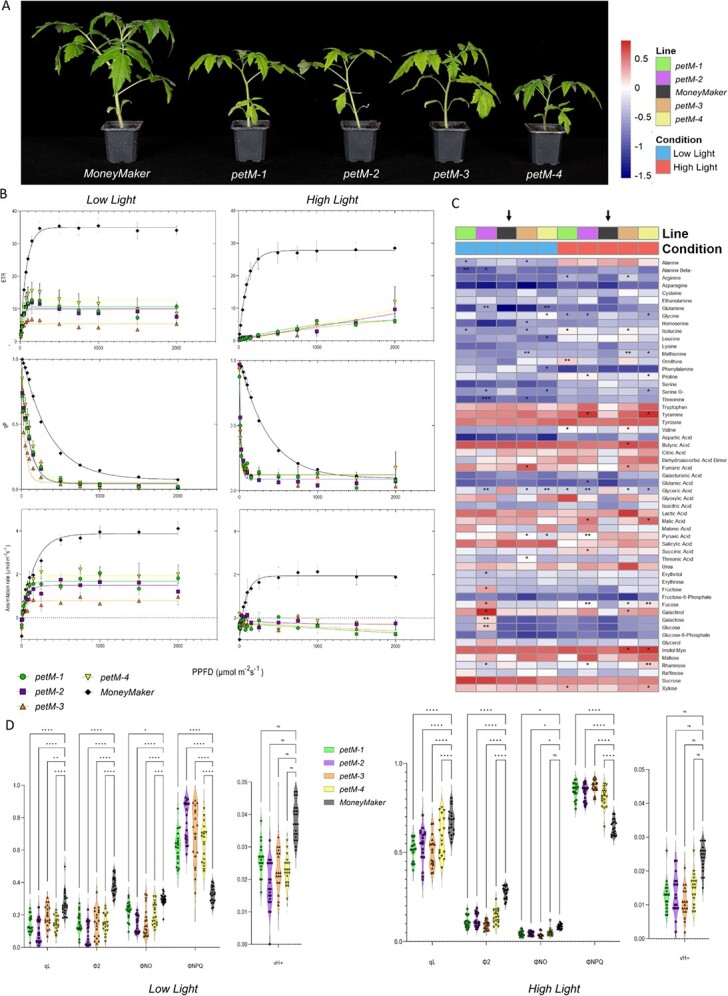
Low Light (LL) and high light (HL) measurements. **(A)** Morphological phenotypes of each independent line during LL and HL evaluation. **(B)** Light curves were generated under LL and HL conditions for each of *petM* and wildtype “MoneyMaker” lines. Parameters indicated are electron transport rate (ETR), photochemical components of chlorophyll fluorescence quenching (q_P_), and CO_2_ assimilation rate. Non-linear one phase regression was performed for each line and parameter, reflected by their color. **(C)** Primary metabolomics changes in *petM* mutants under LL and HL conditions. Colors indicate different mutant lines and “MoneyMaker”. For the statistics, t-tests were performed for each mutant line against “MoneyMaker” under each growth condition, respectively. Significance levels are indicated as follows: ^*^ <0.05, ^**^ <0.01 and ^***^ <0.001. The arrow highlights the reference line, “MoneyMaker”. **(D)** Chlorophyll-*a* fluorescence and absorption measurements over LL and HL conditions. Depicted parameters include q_L_, Φ_2_, Φ_NO_, Φ_NPQ_, and v_H+_. Different lines are indicated in their respective color. The significance levels are depicted as ^*^ <0.05, ^**^ <0.01, ^***^ <0.001 and ^****^ <0.0001, while ns refers to non-significant.

## Results

### Physiological and phenotypic characterization of PetM under low and high light conditions

The PETM subunit of the CB6F was previously reported to play an essential role for CB6F complex accumulation in *N. tabacum* as well as in *A. thaliana* [23, 24]. The RNAi and T-DNA insertion lines, were demonstrated to harbor necrotic leaves and impaired autotrophic growth caused by the malfunction of the chloroplastic electron transport chain, respectively. Initially, due to the reported essential role in Nicotiana and Arabidopsis, its function seemed to be conserved across the plant kingdom. However, in horticultural important fruit crops, namely tomato, the role of the PetM gene has not yet been investigated. To assess its role and further characterize the function, CRISPR/Cas knockout lines in *S. lycopersicum* cv. “*MoneyMaker*”(*WT*) were generated and metabolic, phenotypic and optical analysis in response to different light intensities. In total, four independent mutant lines were selected after screening for further analysis (Figure 1, D). Two of the lines, *petM-1* and *petM-3* showed similar editing sites while the other two lines (*petM-2* and *petM-4*) have unique edits (Figure 1, D). In all mutants, the stop codon occurs upstream of the PetM domain, leading to its absence in the protein sequence. To show there are no off-target effects, we list the highest five off-target regions are listed in Table S1 and the highest-scoring site among them is sequenced (Figure S2).

Initially, mutant lines showed basal autotrophic growth with strong photoinhibition early during the cultivation at 150 μmol photons m^−2^ s^−1^ (Figure 1, C), similar to the previously reported phenotype in *A. thaliana* [24]. To avoid photoinhibition, plants were shifted to low light (LL) conditions at 50 μmol photons m^−2^ s^−1^. Following this, plants displayed a marked recovery yet still displayed delayed autotrophic growth (Figure 2, A). At this stage, fluorescence, absorbance and gas exchange measurements were recorded and leaves harvested for metabolic profiling. In addition to LL measurements, plants were expose for one day to high light (HL) conditions at 800 μmol photons m^−2^ s^−1^, and same measurements were performed as described above (Figure 2, B, C, D). Light response curves were generated across several defined photosynthetic photon flux densities (PPFDs). ETR, photochemical quenching (q_P_) and assimilation rate are plotted against the PPFDs under both light condition for each tomato line (Figure 2, B). Increasing PPFD led under LL condition to significantly decreased electron transport rates in mutant lines compared to *WT*, while overall comparison to HL condition highlights a lower saturation point across all lines caused by photoinhibition. The change of ETR in *WTs* under both condition is rather marginal. In the case of q_P_, under both LL and HL conditions, the mutants showed a significant reduction compared to *WT*, with stronger reduction under HL. These observations were associated positively and negatively with the quantum yield of Photosystem II (Φ_2_) and the yield for dissipation by downregulation (Φ_NPQ_), respectively. Concurrently, the steady-state rate of the photon flux (v_H+_) was statistically invariant between genotypes (Figure 2, D). With regard to the assimilation rate, clear difference could be observed between mutant lines and *WT*. However, upon the shift to HL condition, all plants displayed strong significant reduction in their assimilation rates, while in mutant lines this lead to photorespiration.

In addition to the fluorescence and infrared gas analysis, metabolite profiling using GC–MS to identify metabolic changes in response to PetM knockout was performed. This provides an additional layer to assess the physiological and photosynthetic responses. In this regard, overall more significant changes in primary metabolites were observed under the HL condition. Across all metabolites and both growth conditions, only glyceric acid under the HL condition showed significant decrease in all mutant lines compared to *WT*. Trends of increase and decrease in metabolite levels were observed for several amino acids, such as tryptophan, and other organic acids and sugars (Figure 2, C), for example, under LL condition, glyoxylic acid was increased, while glyceric acid and asparagine were decreased. More changes were observed following the shift to HL, for instance amino acids, such as lysine, arginine, asparagine, methionine, isoleucine, valine, proline, and tyramine were upregulated, while tryptophan and serine were downregulated (Figure 2, C). For organic acids, glyoxylic acid, succinic acid, shikimic acid and citric acid were increased, whereas malic acid and pyruvic acid were downregulated. As for sugars, rhamnose and fucose were upregulated, while trehalose was downregulated.

### Growth and metabolite composition of *petM* mutant leaves under glasshouse conditions

**Figure 3:**
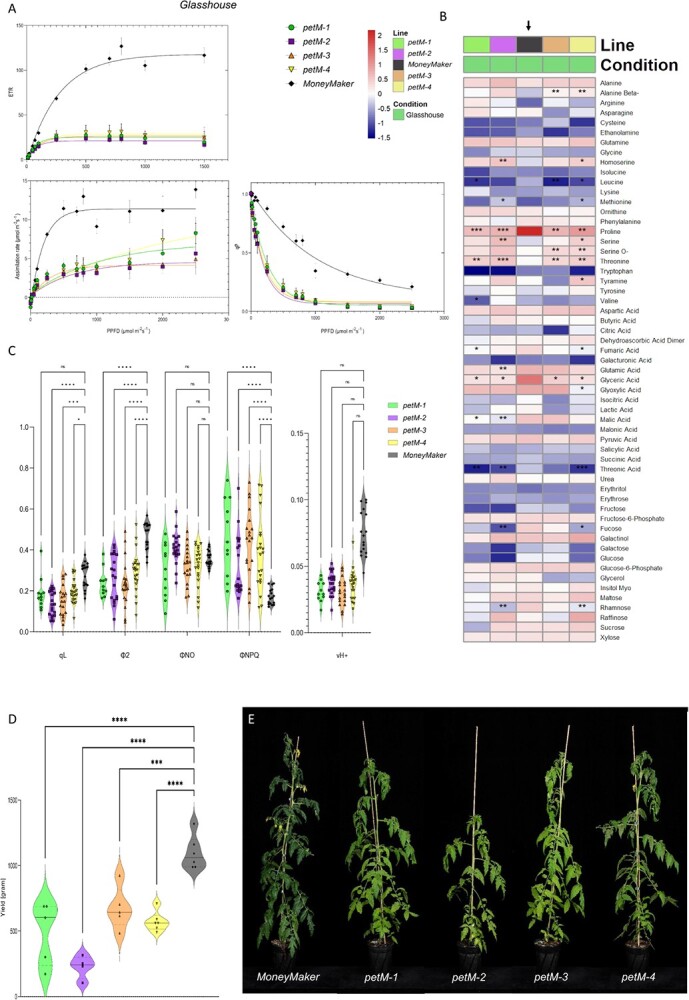
Glasshouse measurements. **(A)** Light curves were generated for each of *petM* and wildtype “MoneyMaker” lines across growth conditions. Parameters indicated are electron transport rate (ETR), photochemical components of chlorophyll fluorescence quenching (q_P_), and CO_2_ assimilation rate. Non-linear one phase regression was performed for each line and parameter, reflected by their color. **(B)** Primary metabolomics changes in *petM* mutants. Colors indicate different mutant lines and “MoneyMaker”. The black arrow highlights the reference line, “MoneyMaker”. For the statistics, t-tests were performed for each mutant line against “MoneyMaker” under each growth condition, respectively. Significance levels are indicated as follows: ^*^ <0.05, ^**^ <0.01 and ^***^ <0.001. **(C)** Chlorophyll-*a* fluorescence and absorption measurements under GH conditions. Depicted parameters include q_L_, Φ_2_, Φ_NO_, Φ_NPQ_, and v_H+_. Different lines are indicated in their respective color. The significance levels are depicted as ^*^ <0.05, ^**^ <0.01, ^***^ <0.001 and ^****^ <0.0001. **(D)** Total fruit weight measurements. Colors indicate different lines. The significance levels are depicted as ^*^ <0.05, ^**^ <0.01, ^***^ <0.001 and ^****^ <0.0001, while ns refers to non-significant. **(E)** Morphological phenotypes of each independent line in the greenhouse.

Following the characterization above, further plants were shifted from LL to glasshouse (GH) conditions to allow vegetative and later reproductive growth to in order to determine the effect of the mutation on fruit yield. As depicted in Figure 3 (E) an additional time point for metabolic and photosynthetic measurements was introduced alongside the LL and HL conditions. The results obtained under this particular condition showed that two and three times higher ETRs in mutant and *WT*, compared to those observed under LL conditions, respectively, (Figure 3, A). Likewise, CO_2_ assimilation rates in *WT* were 2.5 times higher (~10 μmol m^−2^ s^−1^), while the rates in mutant lines were around 2.5–5 μmol m^−2^ s^−1^ (Figure 3, A), suggesting a photosynthetic activity in mutants that is sufficient to maintain its basal functions. In term of photochemical quenching, and the other quenching components, similar patterns were observed to HL, namely we observed increased q_P_, decreased Φ_NPQ_ and stable yield of other non-photochemical losses (Φ_NO_). In addition, no changes in v_H+_ were observed (Figure 3, C). The most significant changes in metabolite levels were observed under the GH condition (Figure 3, B). While under the previous condition, only glyceric acid was significantly downregulated, the levels of proline and threonine were down- and upregulated, respectively, in addition to the downregulation of glyceric acid across all mutant lines under GH conditions (Figure 3, B). Further, multiple other changes were observed, with a plethora of downregulated primary metabolites, such as the organic acids shikimic acid, aconitic acid, maleic acid, malic acid and fumaric acid. Alongside, the sugars rafinose, trehalose, rhamnose and fucose were downregulated. Furthermore, the amino acids, leucine and tryptophan were likewise downregulated, while β-alanine, tyrosine and serine levels were upregulated under GH conditions.

### Composition of the photosynthetic apparatus

**Figure 4:**
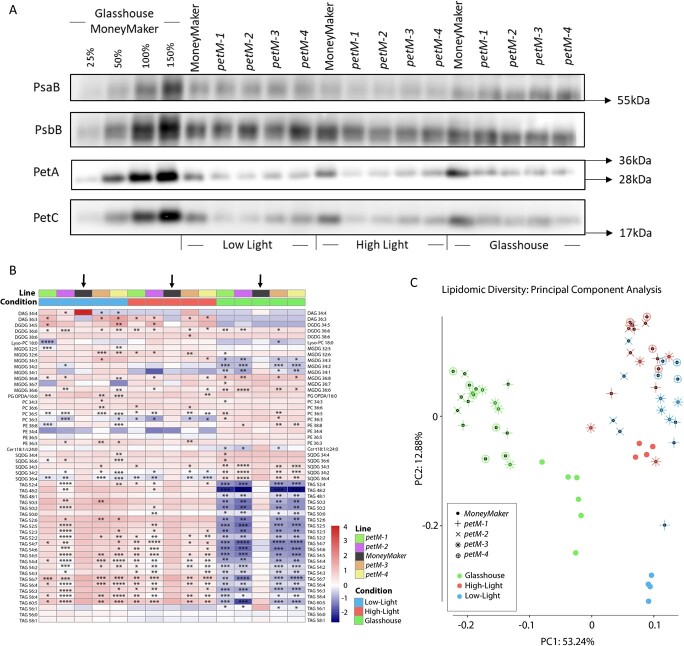
Immunoblotting and lipidomic shifts in *petM* mutants. **(A)** Immunoblot analysis of PetM mutants under different growth conditions. Thylakoids samples equivalent to 1 μg of chlorophyll were loaded and separated in a 10% Tris/Tricin gel and probed with anti-PsaB, anti-PsbB, anti-PetA and anti-PetC antibodies. Glasshouse MoneyMaker was loaded in a dilution series of 25%, 50%, 100% and 150%, respectively. **(B)** Lipidomics content was determined across all growth conditions for each lines. The heatmap indicates lipid contents across growth condition and lines. Different colors reflect different lines and growth conditions, respectively. For the statistics, t-tests were performed for each mutant line against “MoneyMaker” under each growth condition, respectively. The black arrow highlights the reference line, “MoneyMaker”. Significance levels are indicated as follows: ^*^ <0.05, ^**^ <0.01, ^***^ <0.001 and ^****^ <0.0001**. (C)** Principle component analysis using lipidomics leaves data across all growth conditions. PC1 and PC2 explain 53.24% and 12.88% of the variation, respectively.

To investigate changes in photosynthetic complex accumulation, immunoblotting against subunits of PSI, PSII and the CB6F was performed. To this end, thylakoids were isolated from each growth condition. Immunoblotting was performed using antibodies against the essential PSI reaction center subunit PsaB, the PSII inner antenna protein CP47 (PsbB), and two essential subunits of the C*b6f* complex, cytochrome f (PetA) and the Rieske protein (PetC). The results clearly indicate similar levels of PsaB and PsbB accumulation across the growth conditions and tomato lines, while lower levels of PetA and PetC were observed in all mutant lines compared to WT, irrespective of growth conditions, highlighting reduced accumulation, and a potential instability, of the CB6F (Figure 4, A).

### Folial lipidomic profiles

In addition to GC–MS metabolite profiling and fluorescence measurements, lipidomic profiling was performed in mutant and *WT* plants in both leaves and fruit tissues across different growth conditions. Considering the morphophysiological difference in mutant plants, elucidating potential changes in carbon-dense storage compounds, namely triacylglycerols (TAGs), as well as photosynthetic membrane components, such as galactolipids, might be of importance, leading to emphasize lipidomic profiling. Principal component analysis (PCA) was performed on lipidomic profiles with the first two principal components (PCs) explaining 66.12% of the variation (PC1: 53.24%; PC2: 12.88%) (Figure 4, C). PC1 is clearly highlighting divergence of growth conditions, LL/HL and GH. This might be explained either by the environmental condition or the developmental stage, which varied dramatically between LL/HL and GH (Figure 2, A and 3, E). This divergence is explained by differences in TAG levels. By contrast, PC2 separates mutants from *WT* under LL and HL. Within each condition, a clear distinction of *WT* plants from the mutants was observed. When compared to *WT,* the lipidomic profiling revealed significant decreases in multiple lipid classes, mainly diacylglycerols (DAGs), monogalactosyldiacylglycerols (MGDGs), digalactosyldiacylglycerols (DGDGs), and sulfoquinovosyldiacylglycerols (SQDGs) (Figure 4, B).

Investigating the carotenoid derivatives involved in the xanthophyll cycle, which play a major role in NPQ, namely zeaxanthin, antheraxanthin and violaxanthin, changes in their levels across the growth conditions were observed. Specifically, antheraxanthin levels were significantly lower in all mutant lines compared to the *WT* under GH and LL conditions, whereas under the HL condition, antheraxanthin was significantly decreased only in *petM-1* and *petM-2* compared to *WT* plants. Violaxanthin levels were significant altered across all lines in both LL and HL conditions, while under GH conditions, only three lines, *petM-1*, *petM-2* and *petM-3*, showed significantly lower levels. Zeaxanthin levels were decreased in all lines across all growth conditions in comparison to their corresponding *WTs*. The de-epoxidation state (DEPS; = 0.5 ^*^ antheraxanthin + zeaxanthin/ (antheraxantin + zeaxanthin + violaxanthin)) under GH were lower in the mutants, while under LL and HL conditions it showed increased values. Other carotenoids, such as α−/β-carotene, lutein, neoxanthin, and pheophytin, were significantly downregulated in most of the *petM* mutant lines across growth conditions (Table 1). The mutants displayed decreased Chl contents—both for Chl *a* and Chl *b* and the total Chl content—across all lines. However, the Chl *a/b* ratio was lower in the mutants under GH conditions but higher in the mutants under LL and HL, when compared to *WT*.

**Table 1:** Relative abundancies of pigments across growth conditions

**Low Light**	*petM-1*	*petM-2*	*MoneyMaker*	*petM-3*	*petM-4*
Neoxanthin	*0.2495 ± 0.1331* ^**^	*0.2947 ± 0.0759* ^**^	*0.5886 ± 0.0494*	*0.3229 ± 0.0946* ^**^	*0.19 ± 0.0485* ^****^
Violaxantin	*0.3414 ± 0.1959* ^**^	*0.4666 ± 0.1245* ^***^	*1.0332 ± 0.125*	*0.4693 ± 0.1678* ^**^	*0.2697 ± 0.0884* ^***^
Antheraxantin	*0.0109 ± 0.0067* ^**^	*0.0086 ± 0.0042* ^***^	*0.0319 ± 0.0033*	*0.0132 ± 0.0096* ^*^	*0.0076 ± 0.0021* ^****^
Chl-*a*	*20.7 ± 8.1899* ^*^	*21.0551 ± 5.6244* ^**^	*37.8429 ± 2.3793*	*21.402 ± 5.6185* ^**^	*14.6953 ± 3.1324* ^****^
Chl-*b*	*4.6588 ± 1.4654* ^*^	*4.8051 ± 1.0603* ^**^	*7.674 ± 0.3114*	*4.9025 ± 0.9437* ^**^	*3.5314 ± 0.5159* ^****^
total Chl	*25.3587 ± 9.6508* ^*^	*25.8602 ± 6.6785* ^**^	*45.5169 ± 2.6715*	*26.3045 ± 6.5618* ^**^	*18.2267 ± 3.641* ^****^
Chl *a/b*	*4.3364 ± 0.4618*	*4.3466 ± 0.2789* ^*^	*4.9287 ± 0.1407*	*4.3265 ± 0.2774* ^*^	*4.1317 ± 0.2996* ^**^
Lutein	*0.3301 ± 0.0993* ^*^	*0.3511 ± 0.0979* ^*^	*0.5431 ± 0.0686*	*0.3883 ± 0.0612* ^*^	*0.3245 ± 0.0624* ^**^
Zeaxanthin	*0.3301 ± 0.0993* ^*^	*0.3511 ± 0.0979* ^*^	*0.5431 ± 0.0686*	*0.3883 ± 0.0612* ^*^	*0.3245 ± 0.0624* ^**^
Pheophytin	*41.8459 ± 6.6766* ^**^	*42.1915 ± 5.1097* ^***^	*67.5476 ± 5.4296*	*49.9525 ± 2.8515* ^**^	*41.0569 ± 7.184* ^**^
α-Carotene	*0.1264 ± 0.0399*	*0.1315 ± 0.0815*	*0.2078 ± 0.0567*	*0.099 ± 0.029* ^*^	*0.0979 ± 0.0391* ^*^
β-Carotene	*0.1946 ± 0.0497* ^*^	*0.1971 ± 0.094*	*0.3094 ± 0.0577*	*0.1844 ± 0.0396* ^*^	*0.1687 ± 0.0477* ^*^
DEPS	*0.495 ± 0.1349*	*0.4375 ± 0.0502* ^*^	*0.3487 ± 0.0249*	*0.4571 ± 0.071* ^*^	*0.5487 ± 0.0952* ^*^
Zea./Viol.	*1.3198 ± 0.8465*	*0.7659 ± 0.1617* ^*^	*0.5278 ± 0.0607*	*0.8874 ± 0.2772* ^*^	*1.3066 ± 0.4985* ^*^
					
**High Light**	*petM-1*	*petM-2*	*MoneyMaker*	*petM-3*	*petM-4*
Neoxanthin	*0.2482 ± 0.0517* ^*^	*0.2039 ± 0.0642* ^**^	*0.5274 ± 0.1291*	*0.3326 ± 0.0468*	*0.222 ± 0.1207* ^*^
Violaxantin	*0.2231 ± 0.057* ^**^	*0.1627 ± 0.0655* ^***^	*0.5295 ± 0.0919*	*0.2862 ± 0.029* ^**^	*0.2332 ± 0.1787* ^*^
Antheraxantin	*0.0042 ± 0.0005* ^*^	*0.0032 ± 0.0012* ^**^	*0.0086 ± 0.002*	*0.0073 ± 0.0033*	*0.005 ± 0.0033*
Chl-*a*	*18.142 ± 2.2509* ^*^	*14.9912 ± 2.7017* ^*^	*32.9219 ± 7.8335*	*19.7259 ± 1.8388* ^*^	*16.8623 ± 6.8392* ^*^
Chl-*b*	*4.3293 ± 0.4187* ^*^	*3.8877 ± 0.5126* ^*^	*6.9033 ± 1.4895*	*4.8111 ± 0.2768*	*4.1509 ± 1.17* ^*^
total Chl	*22.4713 ± 2.6186* ^*^	*18.8789 ± 3.2078* ^*^	*39.8252 ± 9.3207*	*24.537 ± 2.0868* ^*^	*21.0131 ± 8.0062* ^*^
Chl *a/b*	*4.1863 ± 0.283* ^*^	*3.8402 ± 0.2087* ^***^	*4.748 ± 0.1543*	*4.0961 ± 0.2032* ^**^	*3.9584 ± 0.5295*
Lutein	*0.3395 ± 0.0215* ^*^	*0.2811 ± 0.0231* ^**^	*0.6098 ± 0.1158*	*0.4001 ± 0.0355* ^*^	*0.3299 ± 0.1171* ^*^
Zeaxanthin	*0.3395 ± 0.0215* ^*^	*0.2811 ± 0.0231* ^**^	*0.6098 ± 0.1158*	*0.4001 ± 0.0355* ^*^	*0.3299 ± 0.1171* ^*^
Pheophytin	*44.2929 ± 6.1455* ^*^	*46.3054 ± 3.5317* ^*^	*59.4505 ± 8.6265*	*51.1537 ± 5.3123*	*43.282 ± 5.0709* ^*^
α-Carotene	*0.0853 ± 0.0131* ^*^	*0.0766 ± 0.02* ^**^	*0.1611 ± 0.0329*	*0.0775 ± 0.0154* ^**^	*0.0866 ± 0.0307* ^*^
β-Carotene	*0.1424 ± 0.009* ^*^	*0.1221 ± 0.0179* ^**^	*0.2412 ± 0.046*	*0.1462 ± 0.0265* ^*^	*0.1403 ± 0.0392* ^*^
DEPS	*0.6008 ± 0.0585*	*0.6336 ± 0.0791*	*0.5338 ± 0.017*	*0.582 ± 0.0266* ^*^	*0.5906 ± 0.1069*
Zea./Viol.	*1.5946 ± 0.3879*	*1.9385 ± 0.7335*	*1.1513 ± 0.0805*	*1.4055 ± 0.1608* ^*^	*1.8694 ± 0.8707*
					
**Glasshouse**	*petM-1*	*petM-2*	*MoneyMaker*	*petM-3*	*petM-4*
Neoxanthin	*0.5783 ± 0.1532* ^***^	*0.5447 ± 0.1129* ^***^	*1.1849 ± 0.1759*	*0.5854 ± 0.2671* ^**^	*0.6704 ± 0.3666* ^*^
Violaxantin	*2.0858 ± 0.3813* ^*^	*2.0271 ± 0.301* ^*^	*2.8331 ± 0.5808*	*1.8814 ± 0.3401* ^**^	*2.194 ± 0.7799*
Antheraxantin	*0.0196 ± 0.0041* ^**^	*0.0192 ± 0.0032* ^**^	*0.0386 ± 0.0091*	*0.0206 ± 0.012* ^*^	*0.0241 ± 0.0088* ^*^
Chl-*a*	*36.2379 ± 5.3875* ^**^	*33.4435 ± 4.0934* ^***^	*53.353 ± 4.5058*	*35.0475 ± 7.9077* ^**^	*36.9546 ± 11.2493* ^*^
Chl-*b*	*7.0339 ± 1.049* ^***^	*6.4089 ± 0.9444* ^***^	*11.1924 ± 1.0379*	*6.8213 ± 1.7598* ^**^	*7.3101 ± 2.385* ^**^
total Chl	*43.2718 ± 6.4338* ^**^	*39.8524 ± 5.0349* ^***^	*64.5455 ± 5.5244*	*41.8687 ± 9.6644* ^**^	*44.2647 ± 13.6259* ^*^
Chl *a/b*	*5.1524 ± 0.057* ^****^	*5.2315 ± 0.1322* ^**^	*4.7706 ± 0.0968*	*5.1648 ± 0.151* ^**^	*5.0833 ± 0.1813* ^**^
Lutein	*0.5095 ± 0.0964* ^***^	*0.4542 ± 0.0823* ^****^	*0.9976 ± 0.1484*	*0.5097 ± 0.2054* ^**^	*0.5707 ± 0.2751* ^*^
Zeaxanthin	*0.5095 ± 0.0964* ^***^	*0.4542 ± 0.0823* ^****^	*0.9976 ± 0.1484*	*0.5097 ± 0.2054* ^**^	*0.5707 ± 0.2751* ^*^
Pheophytin	*34.0908 ± 3.6407* ^*^	*30.6115 ± 2.4741* ^**^	*49.9084 ± 10.7073*	*33.784 ± 8.951* ^*^	*32.9392 ± 9.1149* ^*^
α-Carotene	*0.1132 ± 0.0188* ^**^	*0.0907 ± 0.0221* ^**^	*0.1595 ± 0.0195*	*0.0996 ± 0.0233* ^**^	*0.1081 ± 0.0318* ^**^
β-Carotene	*0.2614 ± 0.0483* ^***^	*0.2443 ± 0.0337* ^****^	*0.4559 ± 0.0488*	*0.2604 ± 0.0812* ^**^	*0.2765 ± 0.1049* ^**^
DEPS	*0.1983 ± 0.009* ^**^	*0.1852 ± 0.0055* ^**^	*0.2615 ± 0.0413*	*0.2129 ± 0.0381* ^*^	*0.2071 ± 0.0217* ^*^
Zea./Viol.	*0.2443 ± 0.0137* ^*^	*0.2233 ± 0.0081* ^*^	*0.3625 ± 0.0849*	*0.2663 ± 0.063*	*0.2517 ± 0.034* ^*^

The significance levels of the p-values are calculated based on student t-test of each mutant line against the reference “MoneyMaker” and are depitceted as follows:

^*^< 0.05

^**^< 0.01

^***^<0.001

^****^<0.0001

The values represent mean values across biological replicates with indication of their stardard deviation. Zea. = Zeaxanthin, Viol. = Violaxanthin, Chl = Chlorophyll and DEPS = de-epoxidation state

### Changes in fruit yield and composition

**Figure 5:**
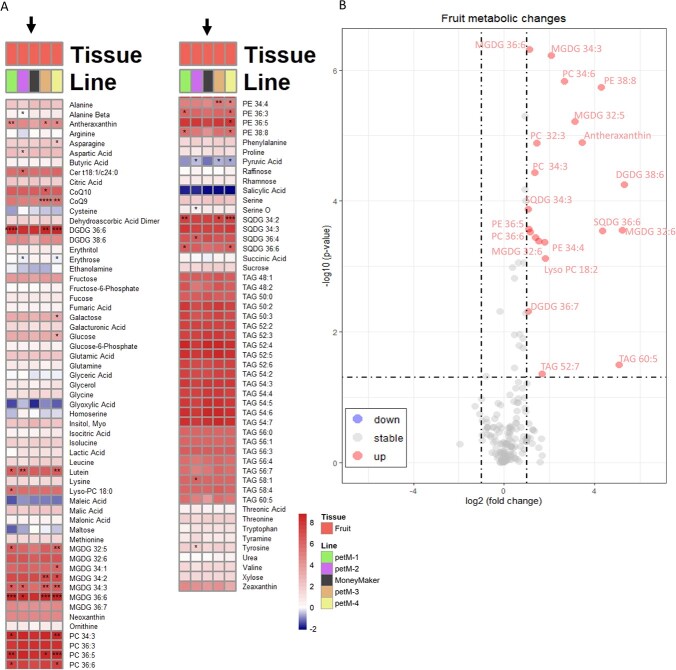
Lipidomic and metabolomic changes in fruits. **(A)** The heatmap indicates lipid and metabolic contents across lines. Different colors reflect different lines. For the statistics, t-tests were performed for each mutant line against “MoneyMaker” under each growth condition, respectively. The black arrow highlights the reference line, “MoneyMaker”. Significance levels are indicated as follows: ^*^ <0.05, ^**^ <0.01, ^***^ <0.001 and ^****^ <0.0001**. (B)** Volcano plot featuring metabolic and lipidomic changes in fruits samples. Blue and red dots highlight significant (raw p-value <0.05) down and up regulated metabolites, respectively, with log_2_ fold changes <−1 and > 1 in combined *petM* mutant comparison against the reference “MoneyMaker”. Significant changed metabolic features with known identities are high with its respective coloration for up and down regulation.

Fruits were harvested to determine the yield performance. In doing so, all mutant lines demonstrated a decreased fruit yield when compared to *WT* (Figure 3, D). Further, metabolic analysis was performed on the pericarp. The combined metabolic and lipidomic data was used to illustrate changes between *WT* and the combined *petM* mutants in a volcano plot (Figure 5, B and Table 2). Multiple lipids in several chemical subclasses, such as MGDGs, DGDGs, phosphatidylcholines (PCs), phosphatidylethanolamines (PEs), TAGs and SQDGs, were significantly upregulated (Figure 5, B and Table 2). In addition, the xanthophyll antheraxanthin was accumulated to significantly higher levels in the mutants. While several other metabolites showed significant changes, the extent of their log_2_ fold changes were lower one. Among these were several lipids, such as PC 32:2, PC 34:5, PC 36:5, PE 36:5, phosphatidylglycerol (PG) 34:3, SQDG 34:3, SQDG 36:4, Lyso-PC 18:2, MGDG 34:2, TAG 58:1, CoQ9 (ubiquinone) and the two pigments, lutein and neoxanthin. In addition, adenine and tyrosine were significantly up-regulated in *petM* mutants. However, these metabolic changes were relatively mild and furthermore only MGDG 34:3, MGDG 36:6, PC 32:3 and PC 34:6 were significantly changing across all individual mutant lines compared to *WT* (Figure 5, A).

**Table 2:** List of significant (p-value <0.05) metabolic and lipidomic changes (log_2_(fold changes) > 1 or log_2_(fold changes) < −1) in ripe fruits pericarp of *petM* mutant compared to “*MoneyMaker”*

**IDs**	**Fold Changes**	**log**_**2**_ **(Fold Changes)**	**Raw p-value**	**LOG**_**10**_ **(p-value)**	
Antheraxanthin	*0.091753*	*3.4461*	*1.29E-05*	*4.891*	↑
DGDG 36:7	*0.47522*	*1.0733*	*0.004927*	*2.3074*	↑
DGDG 38:6	*0.025194*	*5.3108*	*5.57E-05*	*4.2538*	↑
Lyso-PC 18:3	*0.2836*	*1.8181*	*0.000753*	*3.1232*	↑
MGDG 32:5	*0.11383*	*3.135*	*6.09E-06*	*5.2153*	↑
MGDG 32:6	*0.026892*	*5.2167*	*0.000281*	*3.552*	↑
MGDG 34:3	*0.23653*	*2.0799*	*5.93E-07*	*6.2273*	↑
MGDG 36:6	*0.45552*	*1.1344*	*4.76E-07*	*6.3221*	↑
PC 32:3	*0.36637*	*1.4486*	*1.31E-05*	*4.8827*	↑
PC 34:3	*0.3905*	*1.3566*	*3.69E-05*	*4.4327*	↑
PC 36:6	*0.38165*	*1.3897*	*0.000365*	*3.4375*	↑
PE 34:4	*0.29051*	*1.7833*	*0.000427*	*3.3697*	↑
PE 36:5	*0.47093*	*1.0864*	*0.000269*	*3.5706*	↑
PE 38:8	*0.050964*	*4.2944*	*1.81E-06*	*5.7412*	↑
PG 34:4	*0.45136*	*1.1476*	*0.000296*	*3.5286*	↑
SQDG 34:3	*0.47384*	*1.0775*	*0.000132*	*3.8779*	↑
SQDG 36:6	*0.049576*	*4.3342*	*0.000289*	*3.5392*	↑
TAG 52:7	*0.31331*	*1.6743*	*0.044164*	*1.3549*	↑
TAG 60:5	*0.029916*	*5.0629*	*0.032066*	*1.494*	↑

↓ = down regulation in *petM* mutants compared to “MoneyMaker”

↑ = up regulation in *petM* mutants compared to “MoneyMaker”

## Discussion

Indeed, the PETM subunit was already identified as a key component in ensuring stable and high levels of electron flow through the linear electron chain [23, 24]. In the immunoblotting experiment, reduced levels of PETA and PETC, which indicates depletion of the CB6F, were shown. Despite this, the experiments prove that in tomato, *petM* KO lines were able to recover and maintain vegetative and reproductive growth (Figure 3, E). Further, the results demonstrated that under LL adaptation, the *petM* mutants showed basal levels in carbon assimilation and were further impaired in their linear electron flow (LEF) and the ratio of Φ_2_ to Φ_NPQ_ originating potentially from the predicted minor PetM allele.

Morphophysiological changes are mostly concurrent with metabolic adjustments. While primary metabolism was not altered holistically, significant changes in lipid profiles were observed, specifically, significant decreases in the levels of all xanthophylls, which are key components of regulating the dissipation of excess excitation energy via the xanthophyll cycle [27]. Overall the ratio of zeaxanthin to violaxanthin in mutant lines is lower under GH condition and higher in LL and HL condition compared to *WT*. Alongside, considering the DEPS as a proxy for NPQ state, under HL and LL, levels are increasing while under GH condition NPQ apparently decreases in the mutants, which is largely in line with the fluorescence measurements. Based on fluorescence measurements, Φ_NPQ_ was increased in *petM* mutants under all growth conditions, this might indicate a disrupted regulation of the dissipation of excess excitation energy, which would yield in increased ROS accumulation. Upon the shift to the HL condition, the reductive effect on q_P_ and CO_2_ assimilation was dramatically enhanced, with overall constant decrease in carotenoids in both, LL and HL conditions, when compared to *WT*. Under all growth conditions, significant decrease in the carotenoid levels was apparent, with both α-carotene derived metabolites, such as lutein and β-carotene derivatives, such as zeaxanthin, antheraxanthin, violaxanthin, and neoxanthin, were significantly down-regulated. On the other hand, data showed that the mutants have a different mechanism of coping with altered xanthophyll portioning under the different growth conditions, e.g. lower DEPS under GH conditions but higher DEPS under LL and HL condition, when compared to *WT*. Taking the changes in the Chl *a/b* ratio into consideration leads to the assumption that this may be the consequence of adjustments in the PSII and light harvesting complex II (LHCII) ratios [28].

To further investigate these finding, the results were compared with previously characterized *PetM* mutants across higher plants and prokaryotes [23, 24, 26]. In the photogenic prokaryote, cyanobacteria *Synechocystis* PCC 6803, knockout mutation of PetM resulted in no changes in *Cb6f* accumulation, while other photosynthetic components, PSI and PSII were reduced, suggests PetM mediated regulation of the stoichiometry of those protein complexes [26]. The results in *Synechocystis* PCC 6803, *A. thaliana* and *N. tobacco* seemed to illustrate different functions between cyanobacteria and higher plants. However, comparing the defined growth conditions, clear differences in the applied light intensities were observed between organisms (*Synechocystis* PCC 6803 20 μmol photons m^−2^ s^−1^ low light; 100 μmol photons m^−2^ s^−1^ high light; *N. tabacum* PetM RNAi induction 300 μmol photons m^−2^ s^−1^, *A. thaliana* after dark germination at 60 μmol photons m^−2^ s^−1^). In addition, PETM protein sequence comparisons were performed between *Synechocystis* and *S. lycopersicum*, which align at the C-terminus that contains the PetM domain, however *Synechocystis* lacks the N-terminal site of the *S. lycopersicum* sequence, due to overall shorter sequence (*Synechocystis* = 35 amino acids; *S. lycopersicum* = 120 amino acids). This N-terminal site in *S. lycopersicum* is predicted to contain an additional glycoside hydrolase, family 7 domain in the transit peptide site. The PETM subunit is structurally located on the surface of the C*b6f*, and interacts with other components of the thylakoid membrane, such as MGDG, DGDG, SQDG, and PG. This would be in line with the overall decrease of galactolipids in the mutant lines. However, it is important to consider that the changes in lipid profiles might also be due to a pleiotropic response originating from the drastic morphological phenotype.

Previously, it was shown that overexpressing the PETC in *A. thaliana* yielded in consequent increased protein levels of PETA, PETB, as well as proteins of the PSI and PSII complexes [29]. Of major potential agronomic interest, the overall biomass and yield were significantly increased. Across the plant kingdom, variations in assimilation as well as respiration rates are reported [30]. However, overexpression in *N. tabacum* neither yielded higher steady-state electron transport rates nor elevated CO_2_ assimilation, highlighting differences between the role of this protein in different species [31] It is well known that the C*b6f* is a major regulator of the chloroplastic electron transport in C3 plants, although Heyno et al. [31] showed that indeed the C*b6f* is not the sole component in limiting electron transport under higher CO_2_ and light intensities in tobacco. In addition to C3 plants, it was shown that overexpressing the C*b6f* removes chloroplastic electron transport limitations and increase C4 photosynthesis in *Setaria viridis* [32]. Here, we showed that the PetM knockout in the C3 crop (e.g. tomato) revealed an impaired photosynthetic activity and reduced yield.

Based on the observations obtained from different experiments, the current study concludes with following assumptions; first, the growth condition dependent changes in Chl *a/b* ratios observed in the mutant to *WT* comparisons suggests different adjustments in the stoichiometry of PSII/LHCII. This also holds true for the de-epoxidation state, which varies under the LL/HL compared to GH condition, indicating potential shifts of photon fluxes towards Φ_2_, Φ_NPQ_, and Φ_NO_. Furthermore, since the steady-state proton flux through the chloroplastic ATP synthase was not altered significantly, it indicates that similar proton gradients are maintained across the lumen and stroma in mutants and *WT*. Recently, the cryo-EM structure of the spinach CB6F was elucidated and emphasizes the binding of 9-cis-β-carotene between the PETM and PETG subunits [19, 20]. Further, in direct proximity to the 9-cis-β-carotene, Sarewicz et al. [20] identified a thylakoid soluble phosphoprotein (TSP9) locating between the Cb6, SubIV, PETG, PETM, and PETN. With the depleting levels of detected β-carotene in *petM* mutants in comparison to *WT*, leads to the assumption that the docking of β-carotene, and potentially TSP9, is disrupted in the complex. TSP9 was previously shown to undergo phosphorylation and interact with photosynthetic complexes, such as LHCII, PSI and PSII, and thereby potentially play a role in state transition [33]. Further, Sarewicz et al. [20] speculated that TSP9 might play a crucial role in regulating the ratio of linear and cyclic electron transport in higher plants, since the PETP, which is exclusively found in cyanobacteria, binds to a similar region, to where TSP9 in higher plants binds to, and was shown to regulate the balance between linear and cyclic electron transport [34]. This observation might thus provide a potential explanation of stoichiometric adjustments, assumed based on changes in Chl *a/b* ratio and de-epoxidation state.

On the other hand, there is also the possibility of these changes arising through transcriptional and translational feedback regulation of the CB6F accumulation. This would be in contrast to the previously demonstrated upregulation of CB6F by overexpression the petC subunit [29]. However, to assess which of these hypotheses holds truth will require further experiments to clarify the role of PETM in the CB6F and indeed within the photosynthetic apparatus as a whole. Further, unlike Arabidopsis, tomato seemingly contains another predicted allele harboring a PetM domain in its peptide sequence based on different reference genomes (ITAG 3.2 and ITAG 5.0). To test that whether any difference in the expression of second PetM gene is present, primers were designed and its expression measured. Based on qRT-PCR expression analysis, stable expression of the second PetM allele across the *WT* and mutants is demonstrated (Figure S3). Nonetheless, knockout of the allele of interest showed a clear diminishment of CB6F accumulation indicated by the strong reduction of either PETA or PETC highlighting its major contribution in stabilizing the CB6F. Assuming the other predicted allele to be functional, it would only serve a minor role, since most of the CB6F is depleting. However, this would have a detrimental impact on the demonstrated results and interpretation, since this might prevent the lethality of PETM knockout as described for the other species. To this end, single and double knockouts would clarify functionality of the other allele and further the potential of allelic complementation.

In summary, the current study demonstrates that the knockout of PetM in *S. lycopersicum,* which showed initial necrotic growth, could be complemented to a certain degree, by adaptive growth under low irradiation, leading to the hypothesis of this subunit playing a major role in stabilizing and maintaining high ETR under elevated light irradiance. In addition, major changes in CO_2_ assimilation, ETR, as well as primary metabolic and lipidomic changes in leaves and fruits are highlighted. In regards to agronomic importance, as showcased, the PetM knockout ultimately results in reduced yield in tomato plants.

## Material and methods

### CRISPR/Cas9 mutant line generation

Two guide RNAs were designed with an approximate distance of 80 base pairs, which were generated and selected using the tool CRISPR-P 2.0 [35] with the *S. lycopersicum* Genome (SL2.50), accounting for low off-target scores. Constructs were cloned following the strategy described in the protocol of Reem and Van Eck [36], in which two Golden Gate cloning steps are used to generate a level 2 vector. The level 2 vector carries two cassettes expressing the guide RNAs, a selection marker, kanamycin, and the Cas9 nuclease.

The final construct was transformed in *Agrobacterium tumefaciens* strain GV2260 for further downstream *S. lycopersicum cv. Money Maker* transformation. Plant transformation was performed by regeneration of cotyledon segments first on sterile media, followed by co-cultivation with *A. tumefaciens* containing the construct. Cali were transferred on different growth promoting media to induce shoot and root development. Shoot meristem tissue of grown plants were transferred trice onto fresh sterile media containing MS, sucrose (2% m/v), and 125 μg/ml Ticarcillin, to eliminate the possibility of false positives originating from residual *A. tumefaciens* persisting within transformants, while genotyping putative transformants for Cas9 presence by PCR. Genomic DNA was extracted from leaf samples as described in [37]. Another PCR was performed to amplify the region flanking 80 bp up- and downstream of the guide RNA target sites. PCR products were sequenced by Sanger sequencing through LGC genomics (LGC Genomics GmbH, Berlin, Germany). Positive transformants were transferred to the greenhouse.

### Growth conditions

All tomato plants were initially sown and cultivated at 150 μmol∙m^−2^∙s^−1^ of light in a (16 hours/8 hours)–(22°C/18°C)–(70%RH/70%RH)–(day/night) cycle. After the early detection of photoinhibition as shown in Figure 1 (C), plants were transferred to a Percival with reduced light intensities to prevent further inhibition. The Percival was set at 50 μmol∙m^−2^∙s^−1^ of light in a (16 hours/8 hours)–(24°C/20°C)–(60%RH/60%RH)–(day/night) diurnal cycle, as referred to low light conditions (LL) onwards. For high light conditions (HL), an additional Percival was set to the same conditions besides a light intensity of 800 μmol∙m^−2^∙s^−1^. Afterward, plants were transferred to the glasshouse (GH) with a (16 hours/8 hours)–(23°C/20°C)–(60% RH/60% RH)–(day/night) diurnal cycle. A histogram providing GH climate data, including light intensity, relative humidity and temperature across the acclimation time is presented in Figure S1. Plants were water several times during the day by an automated drip irrigation system. Additional treatment included fertilizer supply at transplanting into the glasshouse and during flowering.

### Photosynthetic measurements

Gas exchange and fluorescence measurements were performed using LI-6400 (LI-COR Bioscience; Lincoln, Nebraska, USA). Parameters were measured by generating light response curves for several photosynthetically active photon flux density (PPFD) with 10% actinic blue light. The flow was set at 300 m∙s^−1^, CO_2_ at 400 ppm as well as leaf temperature at 23°C. Plants were adapted at 50 μmol∙m^−2^∙s^−1^ of light for the initial LL measurements. Afterward, additional HL measurements were taken after one day of exposure to 800 μmol∙m^−2^∙s^−1^ of light. Finally, further measurements were taken in the greenhouse (GH) after 4 weeks of transfer. The average measured PPFD in the light period during GH acclimation was approx. 130 μmol∙m^−2^∙s^−1^ (Figure S1). For each date of measurement, dark-adapted fluorescence measurements were taken at midnight.

Additionally, besides the gas exchange measurements, photosynthetic traits, such as the quantum yield of photosystem II (Φ_2_), the fraction photosystem II centers, which are in an open state (q_L_), the ratio of incoming light lost via non-photochemical quenching (Φ_NPQ_) and via non-regulatory processes (Φ_NO_) and the steady state proton flux of the chloroplast ATP synthase (v_H+_) using the MultispeQ [37, 38]. Multispeq measurements were taken using the Protocol “Photosynthesis Rides 2.0”.

### Metabolic characterization

Metabolite analysis for lipid and primary metabolites was performed using a phase-separation-based extraction protocol [39–41]. Fully developed leaves were harvested in the light period and snap-frozen under liquid nitrogen in LL, HL and GH cultivation conditions at a vegetative state. For fruits, ripe fruits were harvested and the pericarp was snap-frozen after removal of the epidermis. In brief, the extraction buffers methyl-*tert*-butyl-ether:methanol (3:1 v/v) and methanol/water (1:3 v/v) were used leading to a two-phase separation. `The extraction buffers contained internal standards, phosphatidylcholine 34:0 and ribitol for the later data normalization. The apolar phase, containing lipids, was dried and used for lipidomic profiling, while the polar phase, containing primary metabolites, was dried and used for downstream derivatization of primary metabolites.

The dried lipid containing extracts were re-suspended in acetonitrile/2-propanol (7:3 v/v) and injected into LC–MS (ThermoScientific). Lipid fractionation was performed on a reversed-phase C_8_ column hold at 60°C with a flow rate of 400 ml/min, and a gradient elution of buffer A, 1% 1 M NH_4_-Acetate and 0.1% acetic acid in water, and buffer B, 1% 1 M NH_4_-Acetate and 0.1% acetic acid in acetonitrile/2-propanol (7:3 v/v). Mass spectra were acquired in positive ionization mode with a pass range of 150–1500 *m/z*.

For primary metabolite analysis, dried polar extracts were linearized by adding 50 μl of methoxyamine hydrochloride in pyridine (30 mg/ml) and shaken at 900 rpm for 2 hours. Afterward, extracts were derivatized using 100 μl N-methyl-N-(trimethylsilyl)trifluoracetamide including FAMES and incubated at 900 rpm for 30 min. Finally, samples were transferred and injected to GC–MS with a 30-m MDN-35 capillary column. Mass selection and peak peaking was performed in Xcalibur (ThermoScientific). In-house libraries were used to determine metabolite identities. Detected peak intensities were normalized first over the internal standards (phosphatidylcholine 34:0 for lipids and ribitol for derivatized metabolites) and afterward over the amount of fresh material used.

### Thylakoid isolation and immunoblotting

The extraction of the thylakoids was performed according to Schöttler et al. [14] from snap-frozen leaf material. Material amount was scaled down to 100 mg snap-frozen leaves. Protein samples normalized to 1 μg of chlorophyll were separated gel electrophoretically as explained in Schägger [42] through a 3% (w/v) and 10% (w/v) stacking and resolving gel, respectively, at 16°C. Nitrocellulose membrane was used for protein transfer at 350 mA for 4 hours, with a constant voltage of 20 V, using transfer buffer containing 39 mM glycine, 48 mM Tris and 20% (v/v) methanol. TBS-T buffer, containing 137 mM NaCl,20 mM Tris–HCl (pH 7.6), Tween 20 0.1% (w/v), in the presence of 0.5% (w/v) bovine serum albumin (Carl Roth GmbH) and 4% (w/v) skimmed milk powder was used for membrane blocking under constant shaking at room temperature for 1 hour. After several washing step in TBS-T, the primary antibody of interest were used to incubate membranes in TBS-T with gentle shaking for 1 hour at room temperature. The primary antibodies used (α-psaB, α-psbB, α-petA, and α-petC) were purchased from Agrisera. Each primary antibody was incubated for 1 hour with several washing steps in between. The appropriate HRP conjugated-secondary antibody (Sigma; diluted 1:50000 in TBS-T) was bind and incubated at room temperature with gentle shaking for 1 hour. ECL Plus western Blotting Detection Kit (GE Healthcare) was used to treat membranes according to the manufacturer's instruction. Finally, the signal was detected in a G:BOX Chemi XT4 (Syngene).

### Yield parameters

At the end of the growing period, fruits were harvested and the total yield was measured by using a scale (Sartorius; Göttingen, Germany).

## Acknowledgements

We would like to thank Dr. Micha Wijesingha Ahchige for guiding and giving advice for the CRISPR/Cas9 vector generation and Dr. Mark A. Schoettler and Dr. Ryo Yokohama for the scientific advices and discussions. Also thanks to Dr. Karin Köhl, the greenhouse team of the Max Planck Institute of Molecular Plant Physiology, for transforming and handling the plants. M.B. appreciates the financial support of the International Max Planck Research School for Molecular Plant Sciences (IMPRS-MolPlant). The research fellowship granted by Conselho Nacional de Desenvolvimento Científico e Tecnológico (CNPq-Brazil) to A.N.-N. is gratefully acknowledged. A.R.F. and S.A. acknowledge the European Union’s Horizon 2020 research and innovation programme, project PlantaSYST (SGA-CSA No. 739582 under FPA No. 664620), and the BG05M2OP001-1.003-001-C01 project, financed by the European Regional Development Fund through the Bulgarian’ Science and Education for Smart Growth’ Operational Programme. S.A. acknowledges the EU Horizon 2020, call HORIZON-WIDERA-2022-TALENTS-01, project NatGenCrop (grant agreement No. 101087091).

## Authors’ contribution

M.B., S.A. and A.R.F. designed the experiment. M.B. performed the experiments and data analysis. M.B., A.R.F. and S.A. wrote the manuscript. A.N.-N. reviewed the manuscript.

## Data availability

The authors declare that all the data supporting the findings of this study are available within the paper and its supporting information files.

## Conflict of Interests statement

The authors declare no conflict of interest.

## Supplementary Data

Supplementary data is available at *Horticulture Research* online.

## Supplementary Material

Web_Material_uhad224
